# New *Clox* Systems for Rapid and Efficient Gene Disruption in *Candida albicans*

**DOI:** 10.1371/journal.pone.0100390

**Published:** 2014-06-18

**Authors:** Shahida Shahana, Delma S. Childers, Elizabeth R. Ballou, Iryna Bohovych, Frank C. Odds, Neil A. R. Gow, Alistair J. P. Brown

**Affiliations:** School of Medical Sciences, University of Aberdeen, Aberdeen, United Kingdom; King’s College London Dental Institute, United Kingdom

## Abstract

Precise genome modification is essential for the molecular dissection of *Candida albicans*, and is yielding invaluable information about the roles of specific gene functions in this major fungal pathogen of humans. *C. albicans* is naturally diploid, unable to undergo meiosis, and utilizes a non-canonical genetic code. Hence, specialized tools have had to be developed for gene disruption in *C. albicans* that permit the deletion of both target alleles, and in some cases, the recycling of the *Candida-*specific selectable markers. Previously, we developed a tool based on the Cre recombinase, which recycles markers in *C. albicans* with 90–100% efficiency via site-specific recombination between *loxP* sites. Ironically, the utility of this system was hampered by the extreme efficiency of Cre, which prevented the construction in *Escherichia coli* of stable disruption cassettes carrying a methionine-regulatable *CaMET3_p_-cre* gene flanked by *loxP* sites. Therefore, we have significantly enhanced this system by engineering new *Clox* cassettes that carry a synthetic, intron-containing *cre* gene. The *Clox* kit facilitates efficient transformation and marker recycling, thereby simplifying and accelerating the process of gene disruption in *C. albicans*. Indeed, homozygous mutants can be generated and their markers resolved within two weeks. The *Clox* kit facilitates strategies involving single marker recycling or multi-marker gene disruption. Furthermore, it includes the dominant *NAT1* marker, as well as *URA3, HIS1* and *ARG4* cassettes, thereby permitting the manipulation of clinical isolates as well as genetically marked strains of *C. albicans*. The accelerated gene disruption strategies afforded by this new *Clox* system are likely to have a profound impact on the speed with which *C*. *albicans* pathobiology can be dissected.

## Introduction

*Candida albicans* is a major opportunistic pathogen of humans. Most healthy individuals carry *C. albicans* as a relatively harmless commensal in the microflora of their oral cavity, gastrointestinal and urogenital tracts. However, the fungus is a frequent cause of mucosal infections (*thrush*) in otherwise healthy individuals, and in severely immunocompromized patients *C. albicans* is able to disseminate throughout the body, causing potentially fatal systemic infections [1], [2]. Therefore, major goals in the field include the dissection of *C*. *albicans* pathobiology as well as the development of more sensitive diagnostic tools and more effective antifungal therapies [3].

The precise mechanistic dissection of *C*. *albicans* pathobiology and drug resistance has depended upon the development of molecular tools that permit the accurate disruption of target genes in this fungus. Several aspects of *C. albicans* biology have slowed progress and demanded the development of *Candida*-specific tools. Specifically, *C. albicans* exists primarily as a diploid, and although haploid forms can now be generated via concerted chromosome loss [4], this fungus does not seem to undergo meiosis to complete a standard sexual cycle [5]–[7]. Therefore, both alleles of a target locus must be disrupted to generate homozygous deletion mutants in *C. albicans*, and ideally, the genetic markers used to select transformants must be recycled to permit the sequential deletion of more than one locus [8]–[11]. As a result, gene disruption in *C.* *albicans* is a relatively time-consuming process. Furthermore, *C.* *albicans* exploits a non-canonical genetic code [12]–[14]. Consequently, specific selectable markers that circumvent the issues associated with usage of the CTG codon have had to be developed for this fungus [8], [13], [15]–[19].

Existing strategies for gene disruption include the exploitation and recycling of the *URA3* marker [8], [10], [11]. These approaches involve the deletion of the first allele in a *C. albicans ura3/ura3* host by targeted integration of a *URA3-*based disruption cassette at the desired locus, and the selection of transformants via uridine prototrophy. Positive selection using 5-fluoroorotic acid (5-FOA) is then required to recycle the *URA3* marker, because the generation of *ura3*- segregants, via homologous recombination between the flanking repeats in these disruption cassettes, is relatively rare [8], [10]. 5-FOA selection for *ura3-* cells is commonly used in model yeasts [20]. However, 5-FOA has been shown to cause chromosomal damage in *C. albicans* [21]. Alternative auxotrophic transformation markers have been developed, such as *HIS1, ARG4* and *LEU2* genes [15], [16], [18], but in most cases these cassettes are not recyclable.

Morschhauser and colleagues [22] addressed the paucity of recyclable marker systems by generating a FLP recombinase-mediated *C. albicans* gene disruption system. In this cassette, FLP expression is regulated by the inducible *SAP2* promoter to mediate site-specific recombination between the FRT sites that flank this *URA3* disruption cassette. The serial use of this system allows the sequential disruption of both target alleles using the *URA3* marker [22]. This system was improved by the addition of a dominant selection marker, *SAT1*, which confers nourseothricin resistance upon *C. albicans* (the *SAT1* flipper): [23]. Shen and co-workers [19] then adapted this FLP-based system by replacing the *SAT1* marker with *NAT1,* which is a codon-optimized *Streptomyces noursei NAT1* gene that also confers nourseothricin resistance. More recently, Morschhauser’s group has described a modified *SAT1* flipper, which was designed to minimize basal FLP expression levels [24]. These recyclable FLP cassettes have proven invaluable tools for the study of genes involved in *C. albicans* pathogenicity. The efficiency of FLP-mediated recombination and marker recycling varies, with reports of 8–40% resolution for the *URA3-*FLP system [22], about 20% for *SAT1*-FLP [23], and more recently, resolution frequencies of up to 100% for *SAT1*-FLP cassettes (Joachim Morschhauser, personal communication).

Recently, we constructed a Cre-*lox*P system for gene disruption and marker recycling in *C. albicans* [25]. Cre catalyses site-specific recombination between *loxP* elements in P1 bacteriophage [26], [27]. This molecular specificity has been exploited through the development of Cre-*lox*P-based recombination tools developed for *Saccharomyces cerevisiae* and mammalian cells [28], [29]. Our *C. albicans* system is analogous to these tools, involving the use of Cre to recycle transformation markers via recombination between flanking *loxP* sites [25]. We constructed a methionine-regulatable *MET3_p_*-*cre* cassette (CAD) and three disruption cassettes with different selectable markers: *loxP*-*ARG4-loxP* (LAL), *loxP*-*HIS1-loxP* (LHL) and *loxP*-*URA3-loxP* (LUL). We were unable to clone *MET3_p_*-*cre* into these *loxP* disruption cassettes because the Cre recombinase encoded by the synthetic, codon-optimized *cre* gene was exceedingly efficient, catalysing self-resolution of *loxP-MET3_p_*-*cre-loxP* cassettes in *E. coli*. Therefore, this Cre-*lox*P system suffers the disadvantage that, in comparison with other gene disruption systems [19], [23], it requires an additional transformation step to introduce the *MET3_p_*-*cre* sequences into *C. albicans* after the two target alleles have been disrupted [25]. However, this Cre-*lox*P system enjoys the advantage of high recombination efficiencies in *C*. *albicans* (>90% marker resolution), thereby circumventing the need to select for resolved segregants [25] and providing the potential to significantly accelerate the gene disruption process.

Here we describe the development of an enhanced Cre-*loxP* toolkit (*Clox*) that exploits the advantages of the old tools while overcoming their disadvantages. The new *Clox* kit facilitates rapid, efficient and flexible gene disruption and marker recycling in *C. albicans,* both for auxotrophic laboratory strains and prototrophic clinical isolates. The construction of a new synthetic, codon-optimized, intron-containing *cre* gene has allowed the inclusion of *MET3_p_*-*cre* within stable, *loxP*-flanked, *Clox* cassettes that carry *URA3* or *NAT1* markers (*URA3-Clox* and *NAT1-Clox*, respectively). These *URA3-Clox* and *NAT1-Clox* cassettes support gene disruption either via the sequential use and recycling of a single marker, or using multiple markers. The efficiency of this *Clox* system permits the accurate generation of resolved homozygous null mutants in less than two weeks, thereby significantly reducing the time required for gene disruption in *C. albicans*. Consequently, the *Clox* system will accelerate functional analysis programmes and provides a platform technology for other forms of genome manipulation in *C. albicans*.

## Results

### The *Clox* kit

**Figure 1:**
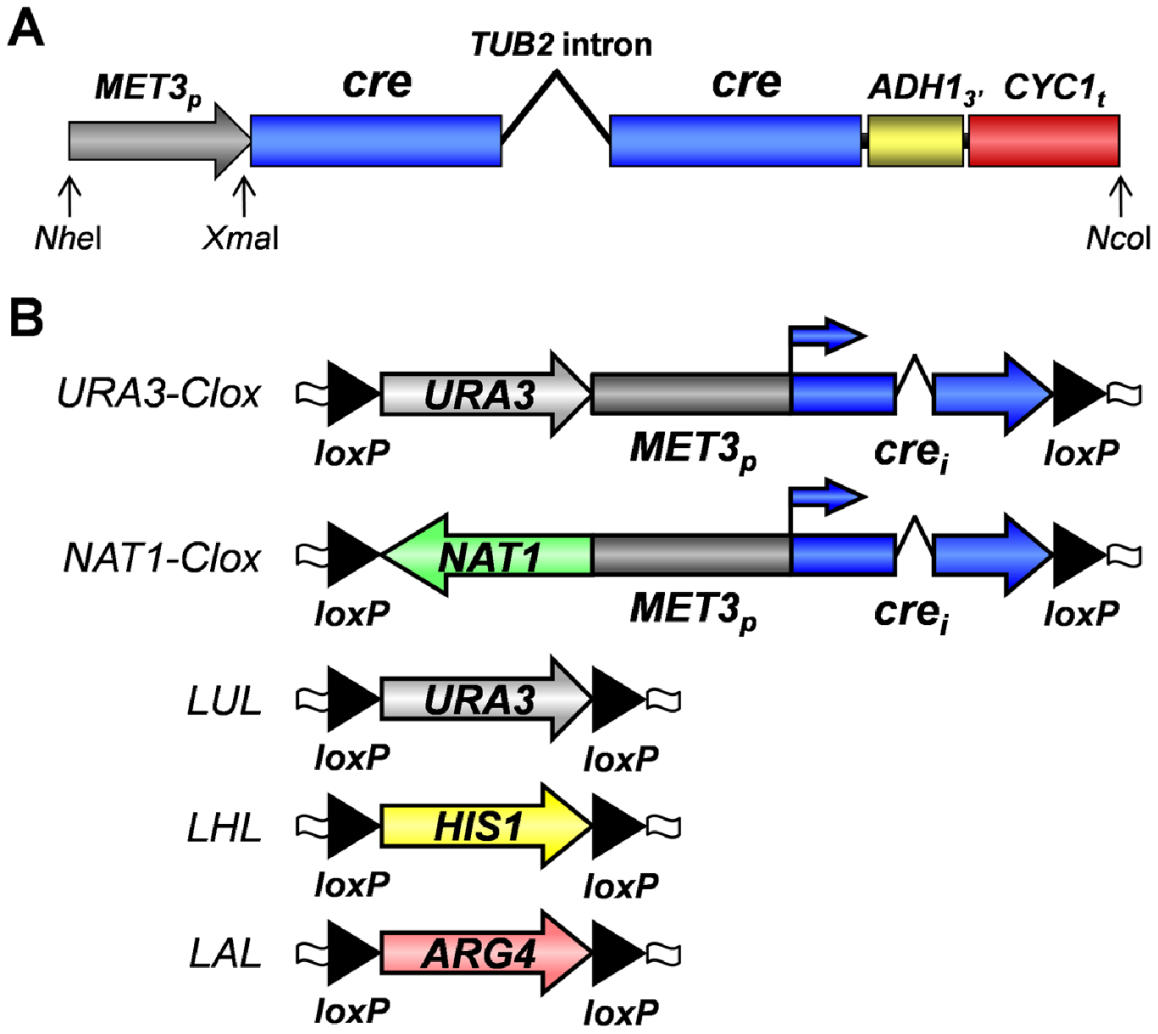
Structure of the synthetic *cre* gene and Clox disruption cassettes. (A) Cartoon illustrating the components of the synthetic *cre* gene including the *CaMET3* promoter (*MET3_p_:* grey), the two codon-optimized *cre* exons (blue), the *CaTUB2* intron, 3′-untranslated sequence from the *CaADH1* gene (yellow), the transcriptional terminator from *ScCYC1* (red), and engineered restriction sites for cloning. The DNA sequence of the synthetic *cre* exons and *CaTUB2* intron is provided in Figure S1. (B) The *Clox* kit. Cartoons illustrating the structures of the *URA3-Clox* and *NAT1-Clox* cassettes (this study) and the *LUL, LHL* and *LAL* cassettes [25]; Black arrows, *loxP* sites; blue arrows, *MET3_p_-cre* transcriptional start sites; open wavey boxes, common PCR priming sites for the disruption cassettes.

The utility of the original Cre-*loxP* system was compromized by the inability to construct stable cassettes carrying *MET3_p_-cre* flanked by *loxP* sites because there was sufficient expression of Cre from *MET3_p_-cre* in *E. coli* to catalyse *loxP* recombination [25]. Therefore, we designed a synthetic intron-containing *cre* gene that would prevent the expression of functional Cre in *E. coli,* whilst permitting the expression of functional Cre in *C. albicans* (Figure 1A). We selected the second intron from the *C. albicans TUB2* gene, because it is relatively short (164 nucleotides) and well characterized [30], [31]. We then introduced two point mutations into the *TUB2* intron to create two in-frame stop codons that would prevent translational read-through of the intron in *E. coli*. This modified *CaTUB2* intron sequence was inserted into the 343 codon *cre* open reading frame such that it interrupts codon 135. The *cre* open reading frame was then codon-optimized, all 18 CTG codons being replaced with preferred leucine codons during this process [25], [32]. Synonymous non-preferred codons were used in places to remove inconvenient restrictions sites. Then a short 3′-untranslated region from *C. albicans ADH1* was added, and transcriptional termination sequences from *S. cerevisiae CYC1* were introduced, because this terminator is well-characterized [33] and is functional in *C. albicans* [34]. Finally, *Nhe*I and *Nco*I sites were designed at the 5′- and 3′-ends of the *cre* gene to facilitate its cloning into the *loxP-URA3-loxP* disruption cassette in the plasmid pLUL2 [25], and a 5′-*Xma*I site inserted to facilitate the subsequent insertion of the *C. albicans MET3* promoter. The structure of this synthetic, intron-containing, codon-optimized *cre* gene is illustrated in Figure 1A, and its complete sequence is presented in Figure S1. The cloning of this synthetic *cre* gene into pLUL2, and the subsequent insertion of *MET3_p_,* generated the plasmid pLUMCL2, which carries the *URA3-Clox* disruption cassette (Figure 1B).

The *URA3-Clox* cassette is suitable for gene disruption in commonly used *C. albicans ura3/ura3* laboratory strains, but is not suitable for the manipulation of prototrophic clinical isolates, which require a dominant selectable marker. Therefore, we replaced the *URA3* sequence in pLUMCL2 with the *NAT1* sequence from pJK863 [19] to create a *NAT1-Clox* cassette in the plasmid pLNMCL (Figure 1B). This cassette permits dominant selection via nourseothrycin resistance.

Previously we constructed a series of vectors to facilitate the construction of control *C. albicans* strains that have the relevant marker genes stably reintegrated into their genomes at the *RPS1* locus (CIp10, CIp20, CIp30: [25]). Therefore, we constructed an analogous plasmid for the reintegration of *NAT1* at *RPS1* (CIp-NAT) (Figure S2). We chose this locus because numerous laboratories have confirmed that the insertion of CIp plasmids at *RPS1* does not affect the phenotype or virulence of *C. albicans* [35].

### The *Clox* Strategy – Multi-marker Disruption

**Figure 2:**
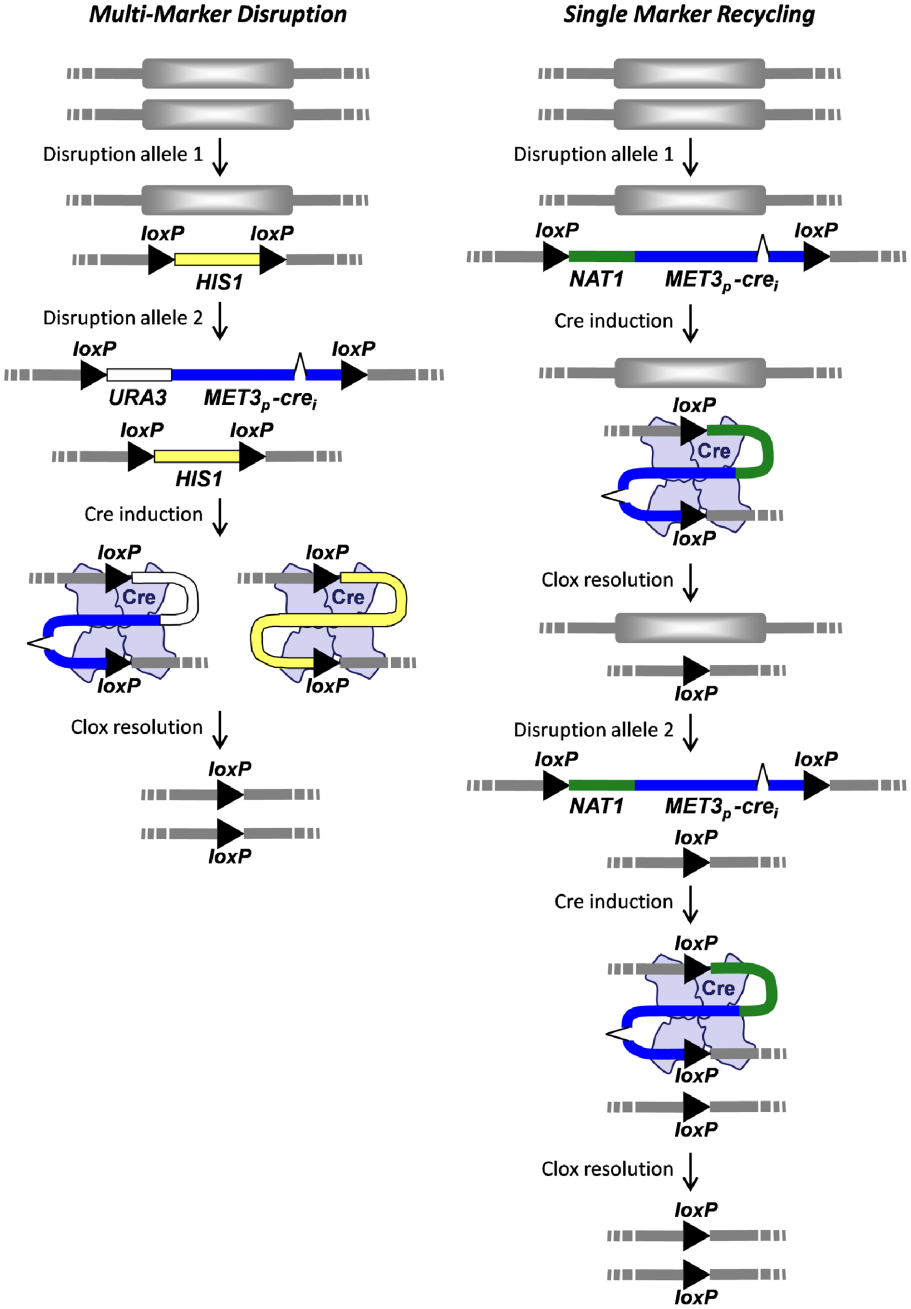
Cartoons illustrating the exploitation of *Clox* cassettes for multi-marker gene disruption and single marker recycling. (see text).

The *NAT1-Clox* and *URA3-Clox* cassettes may be used alone or in combination with existing cassettes that carry alternative auxotrophic markers (Figure 1B). Hence the *Clox* cassettes are suitable for gene disruption in *C. albicans* using both multi-marker disruption and single marker recycling strategies (Figure 2).

**Figure 3:**
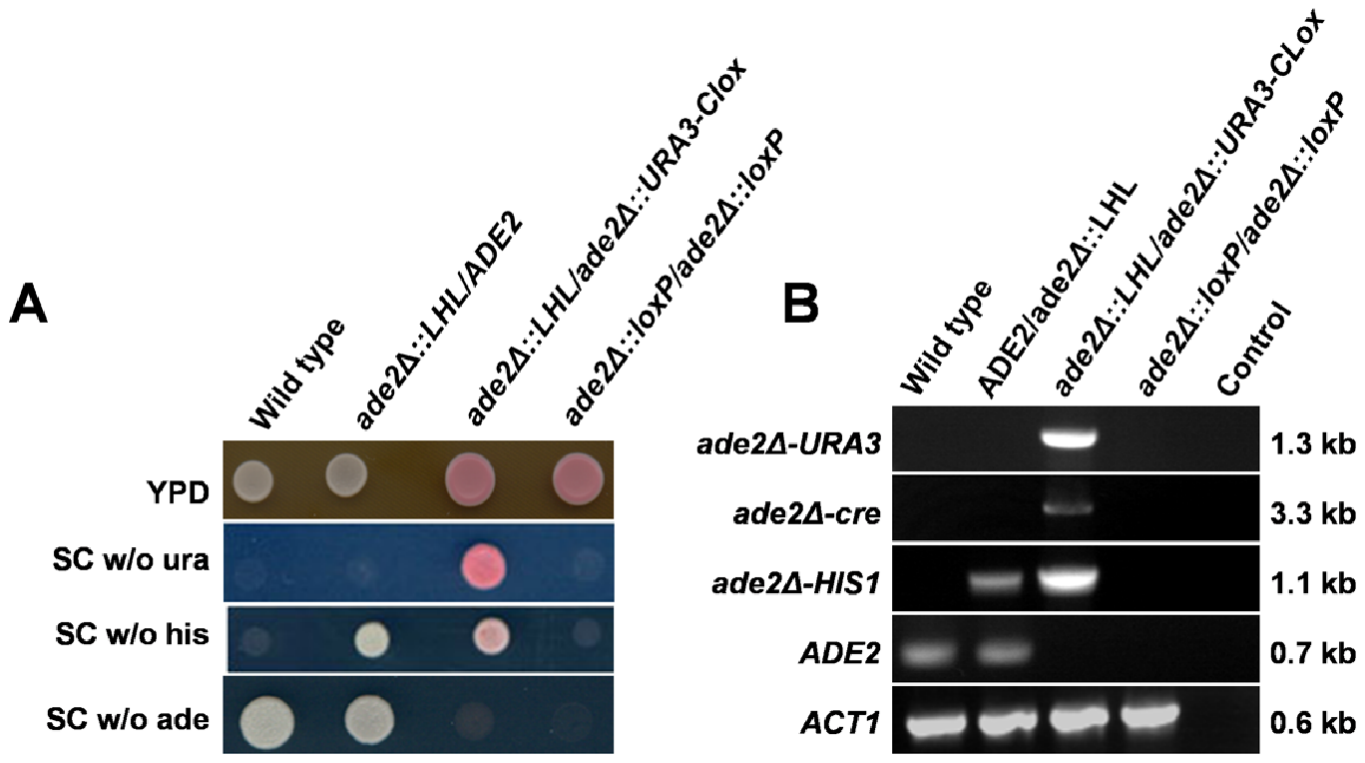
Validation of *Clox* cassettes for multi-marker gene disruption. The *LHL* and *URA3-Clox* cassettes were used to generate a homozygous *ade2Δ/ade2Δ* mutation in *C. albicans* RM1000. (A) Confirmation of the expected auxotrophic requirements for wild type (*ADE2/ADE2*), heterozygous (*ade2Δ::LHL/ADE2*), unresolved homozygous (*ade2Δ::LHL/ade2Δ::URA3-Clox*) and resolved homozygous mutants (*ade2Δ::loxP/ade2Δ::loxP*). Growth media are specified on the right: w/o, without a specific supplement. (B) PCR confirmation of the genotypes for these mutants using primers specific for each allele (specified on the left of each panel). PCR product lengths are given on the right of each panel. *ACT1* was used as a positive control, and a no-DNA control was included (Control).

To validate the multi-marker disruption strategy (Figure 2), we used the LHL (*loxP-HIS1-loxP*) and *URA3-Clox* cassettes (Figure 3) to generate a homozygous *ade2/ade2* null mutation in *C. albicans* RM1000 (*his1- ura3-*: Table 1). The first *ADE2* allele was disrupted by targeted integration of an *ade2Δ::LHL* cassette. The resultant His+ (*ADE2/ade2Δ::LHL*) strain was then transformed with an *ade2Δ::URA3-Clox* cassette to generate a His+ Uri+ (*ade2Δ::LHL/ade2::URA3-Clox*) strain. At each stage, transformants were selected on medium containing methionine and cysteine to repress *MET3_p_-cre* expression. Before marker recycling, transformants were single-celled on fresh medium containing methionine and cysteine to remove untransformed background cells. *MET3_p_-cre* cassettes are stably maintained in the *C. albicans* genome as long methionine and cysteine are present to repress the *MET3* promoter. Then Cre resolves *loxP-*containing cassettes extremely efficiently once this repression is released [25]. Therefore it was important to maintain transformants in the presence of methionine and cysteine. Selecting for *URA3* and *HIS1* transformants when *MET3_p_-cre* is derepressed led to the generation of non-resolvable mutants, essentially because this selects for *C. albicans* segregants that either express non-functional Cre or carry aberrant *loxP* sites.

**Table 1:** Strains used in this study.

Strain	Genotype	Source
SC5314	Clinical isolate	[65]
RM1000	*ura3Δ::imm434/ura3Δ::imm434, his1Δ::hisG/his1Δ::hisG*	[15]
CClox101	RM1000 plus *ADE2/ade2Δ::LHL*	this study
CClox102	RM1000 plus *ade2Δ::URA3-Clox/ade2Δ::LHL*	this study
CClox103	RM1000 plus *ade2Δ::loxP/ade2Δ::loxP*	this study
CClox104	SC5314 plus *ADE2/ade2Δ::Nat1-Clox*	this study
CClox105	SC5314 plus *ADE2/ade2Δ::loxP*	this study
CClox106	SC5314 plus *ade2Δ::Nat1-Clox/ade2Δ::loxP*	this study
CClox107	SC5314 plus *ade2Δ::loxP/ade2Δ::loxP*	this study
CClox108	RM1000 plus *GSH2/gsh2Δ::URA3-Clox*	this study
CClox109	RM1000 plus *GSH2/gsh2Δ::loxP*	this study
CClox110	RM1000 plus *gsh2Δ::URA3-Clox/gsh2Δ::loxP*	this study
CClox112	RM1000 plus *gsh2Δ::loxP/gsh2Δ::loxP*	this study

Having selected His+ Uri+ cells, and confirmed their Ade- status, Cre-mediated recombination was induced by derepressing *MET3_p_-cre* expression. Cells were grown for 4 h at 30°C in SC broth lacking methionine and cysteine and supplemented with adenine, histidine and uridine. Cells were then plated on the same medium. As before [25], over 90% resultant colonies were auxotrophic for uridine and histidine. Hence there was no need to select for resolved (*ura3-*) segregants with 5-FOA. The loss of *HIS1* and *URA3* sequences from these segregants (i.e. the resolution of the LHL and *URA3-Clox* cassettes) was demonstrated by diagnostic PCR (Figure 3B), confirming the functionality of the intron-containing *MET3_p_-cre* gene in *C. albicans*. The strains generated at each stage of the gene disruption process displayed the expected auxotrophic requirements (Figure 3A), and their genotypes were confirmed by diagnostic PCR (Figure 3B).

### The *Clox* Strategy – single Marker Recycling

**Figure 4:**
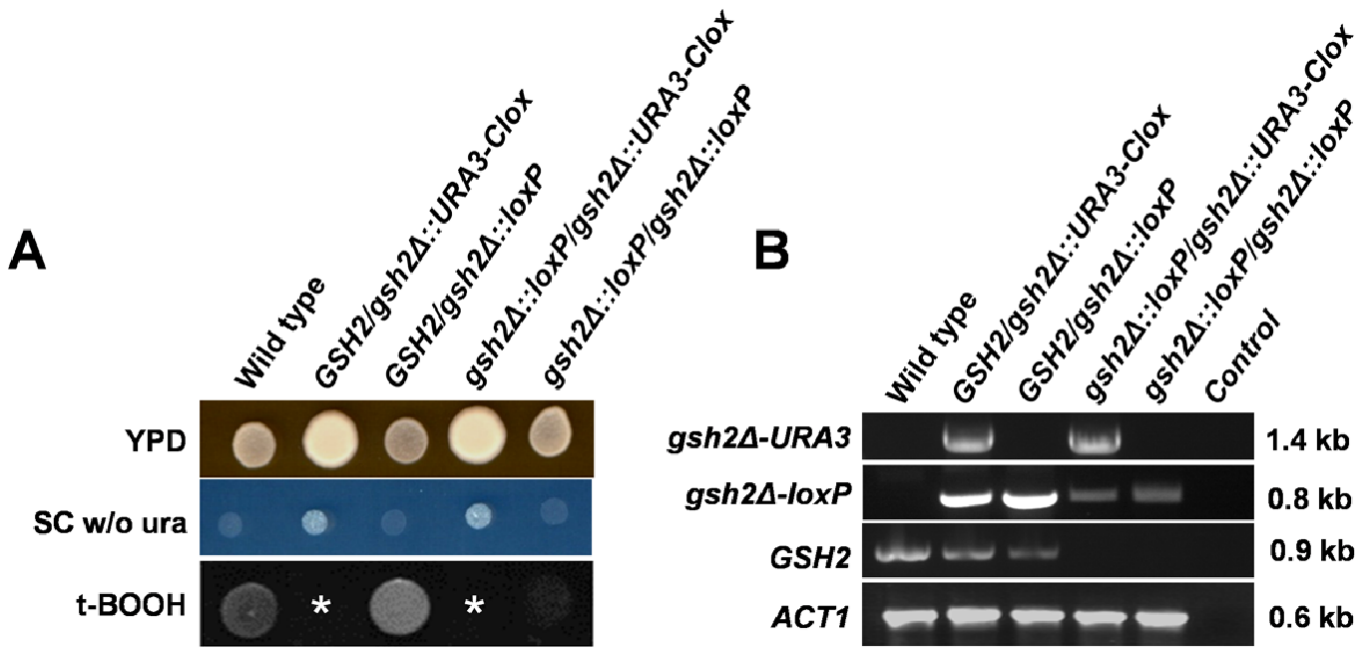
Validation of *URA3-Clox* for single marker recycling. *URA3-Clox* was used to generate a homozygous *gsh2Δ/gsh2Δ* mutation in *C. albicans* RM1000 via single marker recycling (Figure 2). (A) Confirmation of the expected phenotypes for wild type (*GSH2/GSH2*), unresolved heterozygous (*GSH2/gsh2Δ::URA3-Clox*), resolved heterozygous (*GSH2/gsh2Δ::loxP*), unresolved homozygous (*gsh2Δ::loxP/gsh2Δ::URA3-Clox*) and resolved homozygous mutants (*gsh2Δ::loxP/gsh2Δ::loxP*). Growth media are specified on the right: w/o, without a specific supplement; *, it was not possible to test the oxidative stress sensitivity of the unresolved *GSH2/gsh2Δ::URA3-Clox* and *gsh2Δ::loxP/gsh2Δ::URA3-Clox* strains because methionine and cysteine interfere with the oxidizing agent, t-BOOH. (B) PCR confirmation of the genotypes for these mutants using primers specific for each allele (specified on the left). PCR product lengths are given on the right. *ACT1* was used as a positive control, and a no-DNA control was included (Control).

To validate the *URA3-Clox* cassette for single marker recycling (Figure 2), we used it to disrupt *GSH2* (*orf19.6404*), which encodes a putative glutathione synthase in *C. albicans* [36]. *C. albicans* RM1000 was transformed with a PCR-amplified *gsh2::URA3-Clox* cassette (Tables 1 and S1). Uri+ transformants (*GSH2/gsh2Δ::URA3-Clox*) were selected on SC lacking uridine and containing methionine and cysteine, and then streaked on the same medium to select single colonies. The *URA3-Clox* cassette was then resolved by culturing transformants overnight in YPD containing uridine and without supplemental methionine and cysteine. The majority (>90%) of the resultant segregants were Uri- (*GSH2/gsh2Δ::loxP*). The second *GSH2* allele was then disrupted by retransforming Uri- cells with the same *gsh2::URA3-Clox* cassette. Uri+ transformants (*gsh2Δ::loxP/gsh2Δ::URA3-Clox*) were selected on SC lacking uridine and containing methionine and cysteine, and then streaked on the same medium to select single colonies. Transformants were then grown in YPD containing uridine to promote *URA3-Clox* resolution, and then streaked on YPD plates (without supplements) to obtain single colonies. Once again, large numbers of Uri- segregants (*gsh2Δ::loxP/gsh2Δ::loxP*) were generated, and 5-FOA selection was not required. The strains generated at each stage of the process displayed the expected auxotrophies and genotypes, the *URA3-*containing strains growing slightly better on YPD lacking uridine (Figure 4). Interestingly, the diagnostic PCR revealed that some *gsh2Δ::URA3-Clox* cells had undergone Cre-mediated recombination even during growth on media containing methionine and cysteine (Figure 4B). Therefore, under these growth conditions, leaky *MET3_p_-cre* expression appears to be sufficient to promote some *Clox* resolution. *C. albicans gsh2Δ/gsh2Δ* cells were sensitive to oxidative stress (Figure 4A), which is consistent with the predicted glutathione synthase activity of Gsh2 [36].

**Figure 5:**
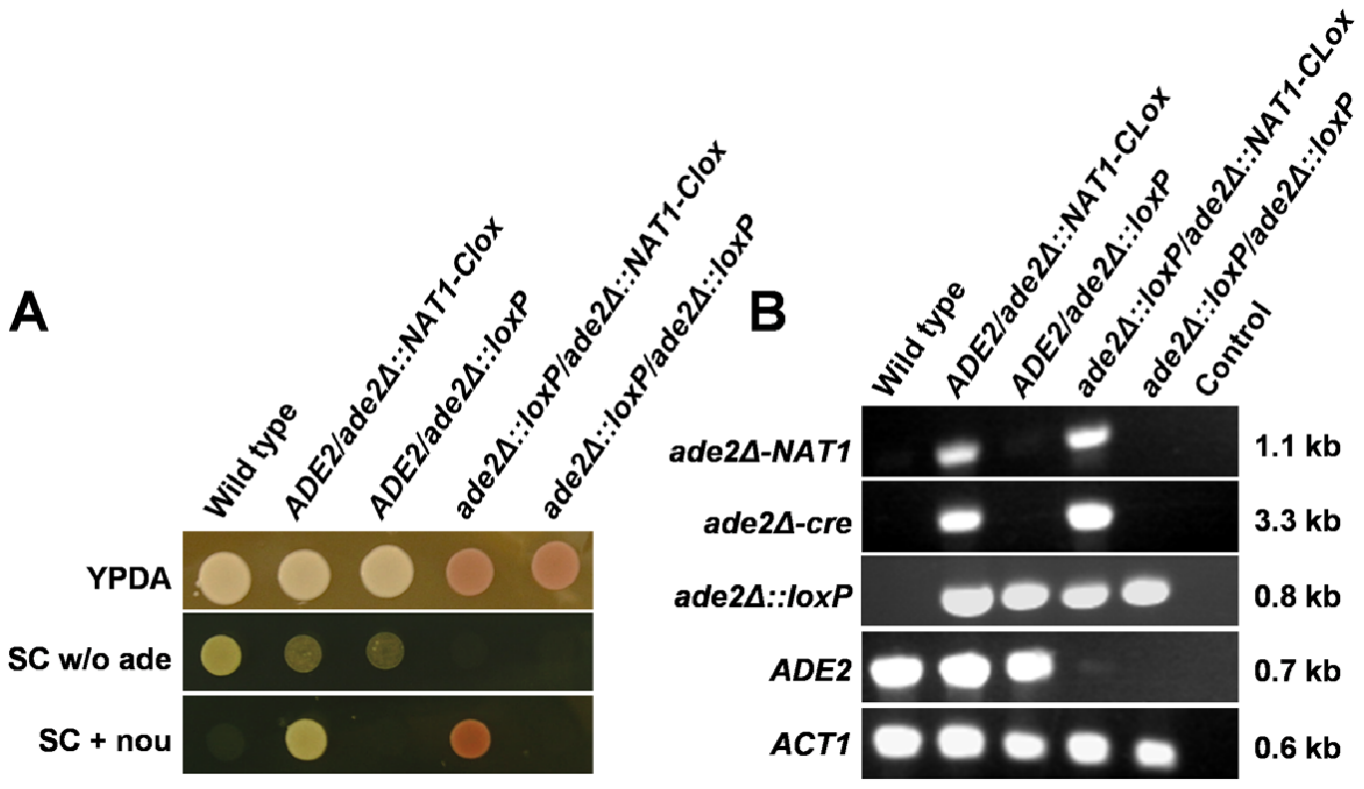
Validation of *NAT1-Clox* for single marker recycling in a prototrophic clinical isolate. *NAT1-Clox* was used to generate a homozygous *ade2Δ/ade2Δ* mutation in *C. albicans* SC5134. (A) Confirmation of the expected phenotypes for wild type (*ADE2/ADE2*), unresolved heterozygous (*ADE2/ade2Δ::NAT1-Clox*), resolved heterozygous (*ADE2/ade2Δ::loxP*), unresolved homozygous (*ade2Δ::loxP/ade2Δ::NAT1-Clox*) and resolved homozygous mutants (*ade2Δ::loxP/ade2Δ::loxP*). Growth media are specified on the right: w/o, without a specific supplement; nou, nourseothricin. (B) PCR confirmation of the genotypes for these mutants using primers specific for each allele (specified on the left). PCR product lengths are specified on the right. *ACT1* was used as a positive control, and a no-DNA control was included (Control).

To confirm that the *NAT1-Clox* cassette can be used to inactivate loci in prototrophic clinical isolates via single marker recycling, we deleted the *ADE2* locus in *C. albicans* SC5314 (Table 1). To inactivate the first *ADE2* allele, cells were transformed with an *ade2::NAT1-Clox* cassette, nourseothricin resistant (Nou^R^) transformants selected on YPD supplemented with nourseothricin, methionine and cysteine, and these transformants restreaked onto the same medium. To resolve the *NAT1-Clox* cassette, purified Nou^R^ isolates were grown overnight in YPD without supplements, and then streaked on YPD plates (without supplements) to obtain single colonies. Nou^S^ segregants were selected (*ADE2/ade2::loxP*), and the second *ADE2* allele was then disrupted with the same *ade2::NAT1-Clox* cassette. Nou^R^ transformants were selected on YPD containing nourseothricin, adenine, methionine and cysteine, and then single-celled on the same growth medium. To stimulate *NAT1-Clox* resolution, Ade- colonies were then grown overnight on YPDA lacking methionine and cysteine. Over 90% of the resultant segregants were Nou^S^ (*ade2::loxP/ade2::loxP*). Once again, the strains generated at each stage displayed the expected auxotrophies and genotypes (Figures 5A and 5B). We observed leaky resolution of the *NAT1-Clox* cassette in *C. albicans* cells grown in the presence of methionine and cysteine, which was similar to the situation for *URA3-Clox* (Figure 4B). These observations confirm the high efficiency of marker recycling with the synthetic intron containing *cre* gene.

## Discussion

The accurate manipulation of specific chromosomal loci is critical for the molecular dissection of microbial development, pathogenicity and drug resistance. Therefore a range of elegant tools have been developed for gene disruption in *C. albicans* [8], [10], [15], [16], [18], [19], [22], [23], . Despite the availability of these tools, gene deletion in *C. albicans* remains a relatively time-consuming process because this fungus is an obligate diploid, apparently unable to undergo meiosis [5]–[7]. Consequently, despite the valiant attempts of a number of groups [18], [38]–[43] we lack a comprehensive collection of homozygous *C. albicans* deletion mutants that is freely available to the academic community. Exciting recent developments suggest that a collection of haploid null mutants could be generated [4], [37], but as things stand *C. albicans* haploids display fitness defects and are unstable [4], [37]. Enhanced gene disruption tools would significantly increase the feasibility of generating a collection of deletion mutants, whether in haploids or diploids. The *Clox* toolkit (Figure 1) offers this enhancement by improving the efficiency with which selectable markers can be recycled, increasing the yields of desired mutants, reducing the number of requisite steps to generate these mutants, and significantly decreasing the time required to generate these mutants.

The recycling of selectable markers in *C. albicans* is desirable for two main reasons. Firstly, a narrow range of auxotrophic markers are available for laboratory strains (*URA3, HIS1, ARG4, LEU2*) [8], [15], [16], [18], and few dominant antibiotic markers can be used in prototrophic clinical strains (*MPA^R^, SAT1/NAT1, HygB*: [19], [23], [44]–[47]). Therefore, the opportunities to dissect multigene families would be severely constrained without marker recycling. Secondly, marker position effects can influence virulence-related phenotypes in *C. albicans* [48]–[51], and therefore markers are generally reintegrated at a standard locus to control for these effects [35], [52], [53]. Initially, marker recycling in *C. albicans* was achieved via homologous recombination between relatively large direct repeats that flank the *URA3* marker gene [8], [10]. However, these *ura3*- segregants arise infrequently, and therefore their isolation depends on positive 5-FOA selection, which is mutagenic [21]. The FLP-system displays markedly improved frequencies of marker recycling that are reported to yield 8–20% of marker-resolved segregants [22], [23], but which can approach 100% resolution (Joachim Morschhauser, personal communication). No selection of Nou^S^ segregants is required, but these smaller colonies must be carefully distinguished from larger Nou^R^ background colonies [23]. Cre-*loxP* also offers extremely high frequencies of marker resolution that can approach 100% in *C. albicans* [25]. However, the utility of the initial system was prejudiced by the inability to clone stable *cre-*containing *loxP-*flanked disruption cassettes in *E. coli* [25]. The construction of an intron-containing *cre* gene has successfully circumvented this problem (Figure 1). The leaky resolution of *Clox* cassettes even in *C. albicans* cells grown on methionine and cysteine, which is a consequence of the extreme efficiency of this system, represents a potential drawback (Figure 4B). Those researchers that need to retain unresolved versions of their mutants might utilise our earlier Cre-loxP system [25], or the current SAT1 flipper [24], which retains the original *Candida*-adapted FLP, a recombinase with lower activity than the mutated ecaFLP gene [54]. However, for most researchers the high Cre efficiency is not an issue, because the desired endpoint is generally the resolved mutant. Furthermore, CIp10, CIp20, CIp30 and CIp-NAT facilitate stable reintegration of the desired markers into resolved mutants [25] (Figure S2).

We have validated the exploitation of *Clox* cassettes for single marker recycling and multi-marker disruption (Figure 2). Single marker recycling demands two cycles of transformation and marker recycling. Given the high yield of correctly resolved *Clox* mutants (>90%) following *MET3_p_-cre* induction ([25]; this study), we find that selections for resolved segregants are not required, and that PCR diagnosis can be left till the end of the disruption process (Figure S3). Therefore, having established the methodology, we proceed directly to the second round of disruption without waiting for PCR confirmation of heterozygous mutant genotypes (retrospective genotyping). The analysis of several segregants from several transformants is generally sufficient to yield the desired homozygous null mutants. As a result we are now able to routinely generate independent, resolved, homozygous null mutants in laboratory strains and clinical isolates within two weeks. The process is even more rapid for the multi-marker disruption strategy which requires only one round of Cre-mediated marker resolution (Figure 2). The notable exception is where inactivation of the target gene confers a significant fitness defect, which necessitates the construction of a conditional mutant [55]–[58]. In principle, one-step gene deletion in haploid *C. albicans* strains should be even faster, although current protocols, which include the cloning of disruption cassettes and flow cytometry to exclude autodiploidized segregants, takes nearly four weeks [37].

The *Clox* cassettes have been tested by other users. Several general points can be made based on their successful construction of over 50 *C. albicans* mutants with *Clox* cassettes (Figure 1). First, off-target integration with *Clox* cassettes does not appear to be a major issue. Off-target integration was rare for those mutants whose genotypes were confirmed by Southern blotting. Also, almost without exception, independently generated mutants have displayed identical or very similar phenotypes. Second, the re-disruption of the first allele is often observed during the second round of disruption when the same PCR primers were used to generate the second disruption cassette, and when it was not possible to impose a double selection (e.g. for LUL and LHL cassettes: Figure 2). However, the desired homozygous null mutant was usually obtained after screening about 20 second round transformants. Furthermore, this issue is circumvented by amplifying the second disruption cassette with primers that target the region deleted from the first allele. Indeed, this approach was successful for all 11 non-essential *C. albicans* loci where this strategy was employed. Third, the *Clox* system does not provide a magical solution to the problems associated with deleting essential loci. Attempts to delete both alleles of 3 *C. albicans* loci that appear to be essential using *Clox* cassettes were unsuccessful. As observed for other systems [10], [15], triploid segregants containing a wild type allele were obtained, rather than the desired homozygous null mutant. We conclude that success rates with *Clox* appear similar to other disruption systems.

These *Clox* trials confirmed a fourth point. Retrospective genotyping of *Clox* mutants is practical for loci without an anticipated phenotype. For the 14 *C. albicans* mutants where this approach was tested, no problems were experienced with retrospective genotyping. Independent homozygous null mutants were successfully generated for 11 of the 14 target loci. For these 11 mutants, 3 independent homozygous mutants were obtained by retrospective screening of 10 second round transformants from each of 5 first round transformants. Regarding the other 3 loci, their apparent essentiality was revealed more quickly by retrospective genotyping. We conclude that the high efficiency of *Clox* marker recycling makes retrospective PCR diagnosis of *C.* *albicans Clox* mutants a feasible option.

We note that the utility of the *Clox* system extends beyond rapid and convenient gene deletion. As for other cassettes [59], [60], the *Clox* system could be adapted to construct fluorescent protein fusions or epitope-tag proteins in laboratory strains or clinical isolates. In principle, *Clox* could also be exploited to engineer large chromosomal deletions [61], or the induction of genetic alterations that allow the analysis of spatial and temporal patterns of gene expression and their role in development [62]. Therefore, the *Clox* system represents a significant step forward in the development of the *C. albicans* molecular toolbox that should empower local and genome-wide analyses of this major opportunistic pathogen of humans.

## Materials and Methods

### Strains and Growth Conditions

*C. albicans* strains used in this study are listed in Table 1. Unless otherwise specified, all strains were grown in YPD [63]. In some cases strains were grown on YPDG (YPD containing 40 µg/ml glutathione) or YPDA (YPD containing 0.01% adenine). SD medium supplemented with auxotrophic requirements or SC medium lacking the appropriate supplement [60] were used to screen *C. albicans* cells transformed with *Clox* disruption cassettes. During all selections for *Clox* transformants, and for all phenotyping assays, media were supplemented with 2.5 mM methionine and 2.5 mM cysteine to repress the *MET3* promoter and minimize Cre-*lox*P mediated recombination. Nourseothricin resistant (Nou^R^) transformants were selected using 200 µg/mL nourseothricin (Werner Bioagents, Jena, Germany). For phenotyping assays, strains were grown overnight at 30°C, 200 rpm in SC medium containing the appropriate supplements plus 2.5 mM methionine and 2.5 mM cysteine. These cells were diluted in sterile water, and 10^4^ cells were spotted onto agar plates, which were then incubated at 30°C for two days before imaging.

### *Clox* Construction

A synthetic, codon-optimized *cre* open reading frame, interrupted by a *C. albicans TUB2* intron at codon 135, was designed *in silico* (Results), constructed by DNA2.0 (Menlo Park, CA, USA) and cloned between the *Nhe*I and *Nco*I sites in pLUL2 [25] to generate pLUCL2. The *CaMET3* promoter region (1336 bp) was then PCR-amplified using Infusion cloning primers Clox-MET3p-F and Clox-MET3p-R (Table S1) and cloned between the *Nhe*I and *Xma*I sites in pLUCL2 in front of the *cre* gene using an In-Fusion HD cloning kit according to the manufacturer’s instructions (Clontech, California, USA) to generate the *URA3-Clox* cassette in the plasmid pLUMCL2. The *URA3* marker in pLUMCL2 was then replaced with the *NAT1* marker to generate the *NAT1-Clox* cassette in the plasmid pLNMCL. *NAT1* was amplified from pJK863 [64] using the primers Clox-NAT1-F and Clox-NAT1-R (Table S1), and then cloned between the *Bpu10I* and *NheI* sites of pLUMCL2 by In-Fusion cloning to create pLNMCL. The sequences of the *URA3-Clox* and *NAT1-Clox* cassettes were confirmed experimentally. The structures of all *Clox* cassettes are illustrated in Figure 1, and their sequences are available in GenBank:

- *URA3-Clox* (*loxP-URA3-MET3_p_-cre-loxP*): GenBank accession number KC999858
- *NAT1-Clox* (*loxP-NAT1-MET3_p_-cre-loxP*): GenBank accession number KC999859
- LAL (*loxP-ARG4-loxP*): GenBank accession number DQ015897
- LHL (*loxP-HIS1-loxP*): GenBank accession number DQ015898
- LUL (*loxP-URA3-loxP*): GenBank accession number DQ015899

### CIp-NAT

CIp-NAT is a *Candida albicans* integrating plasmid (CIp) based on pJK863 [19], a kind gift from Julia Kohler. pJK863 carries a FLP-recyclable, codon-optimized *NAT1* gene. To create CIp-NAT, the *RPS1* targeting sequence, including the *Stu*I linearization sites, was amplified from CIp10 [52] using the primers RPS1-NAT1-F and RPS1-NAT1-R (Table S1). The resulting PCR product was cloned between the *Sac*II and *Sac*I sites in pJK863, thereby generating CIp-NAT (Figure S2). The plasmid was sequenced from m13F to m13R and the data were deposited in GenBank under accession number KJ174065.

### Gene Disruption Using *URA-Clox* and *NAT-Clox*

The *URA3-Clox* and *NAT1-Clox* cassettes were PCR-amplified using Extensor master mix (Thermo scientific; MA, USA) with chimeric primers, the 5′-ends of which represented short (90–100 bp) flanking regions of homology to the target locus [59], and the 3′-ends of which hybridized to the PCR priming sites common to all *Clox* cassettes (Table S1). The resulting PCR products were used to transform *C. albicans* [64]. Transformants were selected on nourseothricin-containing or uridine-lacking medium that also contained 2.5 mM methionine and 2.5 mM cysteine to repress *MET3_p_-cre* expression and inhibit marker resolution. Fresh Nou^R^ and Uri+ transformants were single-celled on fresh medium containing 2.5 mM methionine and 2.5 mM cysteine, and if necessary, their genotypes confirmed by diagnostic PCR with the primers described in Table S1.

### Cre-mediated Marker Resolution

After streaking for single colonies, *C. albicans* transformants were grown overnight in 10 ml SC medium that contained 2.5 mM methionine and 2.5 mM cysteine (to repress *MET3_p_-cre* expression) and maintained marker selection (i.e. lacked uridine or contained nourseothricin). Cells were harvested by centrifugation, washed twice in sterile H_2_O, and resuspended in 10 ml SC that lacked methionine and cysteine (to induce *MET3_p_-cre* expression) and without marker selection (i.e. lacked nourseothricin or contained uridine, and if necessary, contained any supplement required to support the new gene knock-out). Cells were incubated in this medium at 30°C for 4 h, and then streaked onto plates containing the same growth medium. The genotypes of the resolved mutants were then confirmed by diagnostic PCR using the primers described in Table S1.

### Oxidative Stress Resistance

The oxidative stress resistance of *C. albicans* control and *gsh2/gsh2* strains was tested by growing the strains overnight at 30°C, 200 rpm in SC medium containing the appropriate supplements, plating 10^4^ cells on YPD containing 1 mM tert-butylhydoperoxide (tBOOH), and incubating the plates at 30°C for two days.

### Ethics Statement

No ethical permissions were required for this work which involved no experimentation involving animals or human samples.

## Supporting Information

Figure S1 **Annotated DNA sequence of the synthetic, codon-optimized, intron containing** ***cre*** **gene.**
(PDF)

Figure S2 **Structure of the CIp-NAT plasmid, for targeting** ***NAT1*** **to the** ***RPS1*** **locus.**
(PDF)

Figure S3 **Protocol for multi-marker gene disruption using** ***Clox*** **cassettes.**
(PDF)

Table S1 **Primers used in this study.**
(PDF)
