# Supplementary material for: New Clox Systems for Rapid and Efficient Gene Disruption in Candida albicans
Source: PLoS One. 2014 Jun 18;9(6):e100390. doi: 10.1371/journal.pone.0100390 (PMC4062495; doi:10.1371/journal.pone.0100390)
Supplement: Figure S1 — Annotated DNA sequence of the synthetic, codon-optimized, intron containing cre gene. (PDF) [file pone.0100390.s001.pdf]

Figure S1. Annotated DNA sequence of the synthetic, codon-optimized, intron containing *cre* gene.
Nucleotide sequence of the *cre* gene with the corresponding amino acid sequence (single letter code); restriction sites, pink letters; *CaTUB2* intron, blue lower case; mutations that introduce in-frame stop codons in the intron, red lower case; *CaADH1* terminator sequence, red upper case; *S. cerevisiae CYC1* terminator sequence, green upper case.

```
NheI                 XmaI
GCTAGCTTACTTATATACCCGGGTATACTCATTAATCAATAATTATGTCTAACTTGTTGACCGTTCACCAAAACTTGCCAGCTTTGCCA
                                            M  S  N  L  L  T  V  H  Q  N  L  P  A  L  P

GTTGATGCTACCTCTGATGAAGTTAGAAAAAACTTGATGGATATGTTTAGAGATAGACAAGCCTTCTCTGAACACACCTGGAAAATGTTG
V  D  A  T  S  D  E  V  R  K  N  L  M  D  M  F  R  D  R  Q  A  F  S  E  H  T  W  K  M  L

TTGTCTGTTTGTAGATCCTGGGCTGCTTGGTGTAAATTGAACAACAGAAAATGGTTCCCAGCCGAACCAGAAGATGTTAGAGATTACTTG
L  S  V  C  R  S  W  A  A  W  C  K  L  N  N  R  K  W  F  P  A  E  P  E  D  V  R  D  Y  L

TTGTACTTGCAAGCTAGAGGTTTGGCTGTTAAAACCATTCAACAACACTTGGGTCAATTGAACATGTTGCACAGAAGATCCGGTTTGCCA
R  P  S  L  Y  L  Q  A  R  G  L  A  V  K  T  I  Q  Q  H  L  G  Q  L  N  M  L  H  R  R  S

AGACCATCTGATTCTAACGCTGTTTCTTTGGTTATGAGAAGAATTAGAAAAGAAAACGTTGATGCTGGTGAAAGAGCTAAACAAGCCTTg
G  L  P  D  S  N  A  V  S  L  V  M  R  R  I  R  K  E  N  V  D  A  G  E  R  A  K  Q  A  L

taggtatagacactgaagaaaaaaaaattctatcattgttatgttgtgatctttgatctttagttgtcgggttaacacctgccaattgga
*     *

tcaatacatcaatcaattaattctaatcttgaaaaaaaaattcattttactaacaatttttcttttatttagGGCTTTCGAAAGAACCGAT
                                                                          A  F  E  R  T  D

GTTTTCGATCAAAGATCCTTGATGGAAAACTCTGATAGATGTCAAGATATTAGAAACTTGGCTTTCTTGGGTATTGCTTACAACACCTTGTTG
F  D  Q  V  R  S  L  M  E  N  S  D  R  C  Q  D  I  R  N  L  A  F  L  G  I  A  Y  N  T  L  L

AGAATTGCTGAAATTGCTAGAATTAGAGTTAAAGATATTTCCAGAACCGATGGTGGTAGAATGTTGATTCACATTGGTAGAACCAAAACCTTG
R  I  A  E  I  A  R  I  R  V  K  D  I  S  R  T  D  G  G  R  M  L  I  H  I  G  R  T  K  T  L

GTTTCTACCGCTGGTGTTGAAAAAGCCTTGTCTTTGGGTGTTACCAAATTGGTTGAAAGATGGATTTCTGTTTCTGGTGTTGCTGATGATCCA
V  S  T  A  G  V  E  K  A  L  S  L  G  V  T  K  L  V  E  R  W  I  S  V  S  G  V  A  D  D  P
```

```
AACAACTACTTGTTCTGTAGAGTTAGAAAAAACGGTGTTGCTGCTCCATCTGCTACCTCTCAATTATCTACCAGAGCTTTGGAAGGTATT
N  N  Y  L  F  C  R  V  R  K  N  G  V  A  A  P  S  A  T  S  Q  L  S  T  R  A  L  E  G  I

TTCGAAGCTACCCACAGATTGATTTACGGTGCTAAAGATGATTCTGGTCAAAGATACTTGGCTTGGTCTGGTCACTCTGCTAGAGTTGGT
F  E  A  T  H  R  L  I  Y  G  A  K  D  D  S  G  Q  R  Y  L  A  W  S  G  H  S  A  R  V  G

GCTGCTAGAGATATGGCTAGAGCCGGCGTTTCTATTCCAGAAATTATGCAAGCTGGTGGTTGGACCAACGTTAACATTGTTATGAACTAC
A  A  R  D  M  A  R  A  G  V  S  I  P  E  I  M  Q  A  G  G  W  T  N  V  N  I  V  M  N  Y

ATTAGAAACTTGGATTCTGAAACCGGTGCTATGGTTAGATTGTTGGAAGATGGTGATTAATTTATACCTAGGTAAGCAAATAGCTAAATT
I  R  N  L  D  S  E  T  G  A  M  V  R  L  L  E  D  G  D  *

ATATACGAATTAATATTATGATTAAGTGTTTACGTGAGTGCGATATTTTTATTACTATCTTATACAGTTGTATATACTCTATAAAATGAG

                                                                                      NcoI
TTGTCTATTAATTAACGCGATGAGTCCCTATTTATTTTTTTATAGTTATGTTAGTATTAAGAACGTTATTTATATTTCAAATTAATTATCCTAGG
```
