# Supplementary material for: New Clox Systems for Rapid and Efficient Gene Disruption in Candida albicans
Source: PLoS One. 2014 Jun 18;9(6):e100390. doi: 10.1371/journal.pone.0100390 (PMC4062495; doi:10.1371/journal.pone.0100390)

Figure S2. Structure of the CIp-NAT plasmid, for targeting *NAT1* to the *RPS1* locus. Note that the *NAT1* marker is present with a FLP-resolvable cassette [19], which permits recycling of this marker if necessary. The *NAT1* flipper cassette (including the *NAT1* marker (NatR), the *SAP2p*-regulated *FLP1* resolvase gene, and the FRT sites [arrow heads]), the *RPS1* sequence (for targeting integration at the *C. albicans RPS1* locus), and *E. coli* plasmid sequences (ColE1, AMP^R marker, m13F) are indicated.

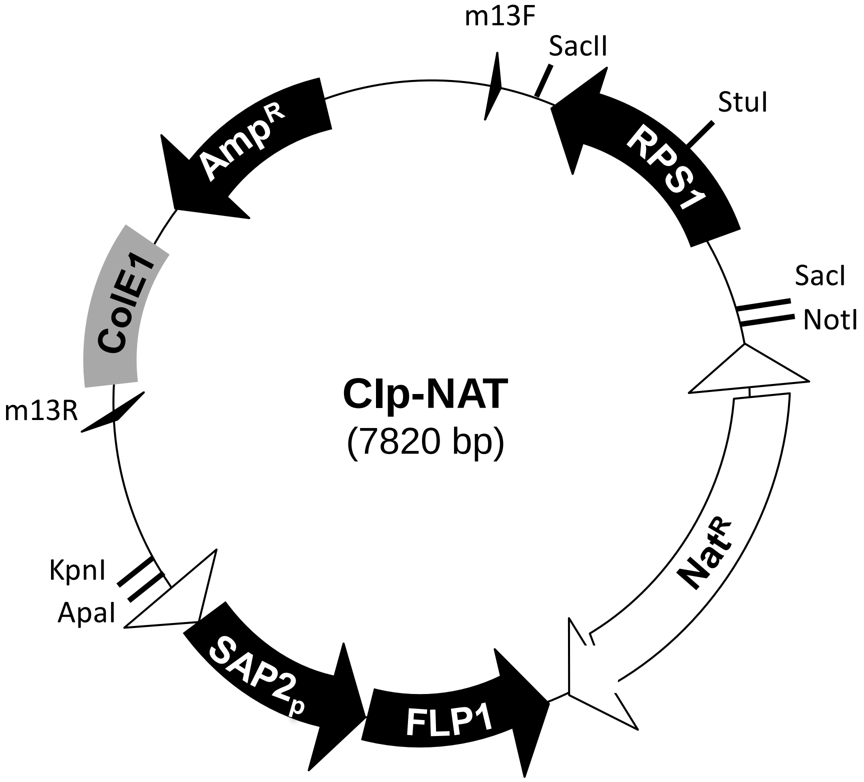

Supplement: Figure S2 — Structure of the CIp-NAT plasmid, for targeting NAT1 to the RPS1 locus. (PDF) [file pone.0100390.s002.pdf]
