# Supplementary material for: New Clox Systems for Rapid and Efficient Gene Disruption in Candida albicans
Source: PLoS One. 2014 Jun 18;9(6):e100390. doi: 10.1371/journal.pone.0100390 (PMC4062495; doi:10.1371/journal.pone.0100390)
Supplement: Figure S3 — Protocol for multi-marker gene disruption using Clox cassettes. (PDF) [file pone.0100390.s003.pdf]

Figure S3: Protocol for multi-marker gene disruption using *Clox* cassettes

PCR amplify *LHL* and *NAT1-Clox* disruption cassettes

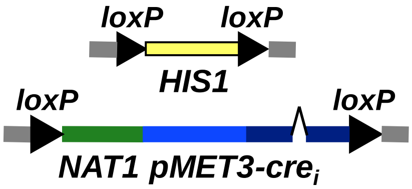

Day 1

Transform *C. albicans* with *LHL,* selecting transformants on SC medium minus histidine.

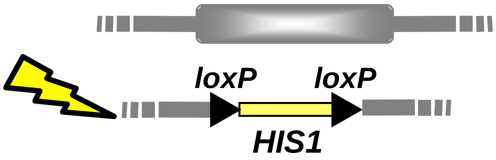

Days 2-5

Restreak cells on SC medium minus histidine
Confirm genotype by PCR

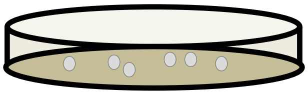

Days 5-7

Transform cells with *NAT1-Clox,* selecting transformants on SC plus nourseothricin, methionine, cysteine, plus supplement(s) necessitated by the gene activation

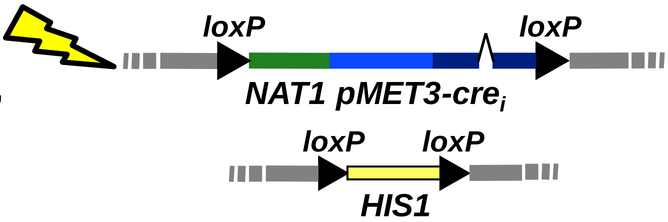

Days 8-11

Restreak cells on SC minus histidine plus nourseothricin, methionine, cysteine, plus supplement(s) necessitated by the gene activation

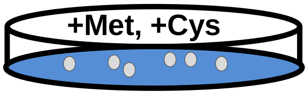

Days 11-12

To induce Cre-mediated recombination, incubate transformants for 4h at 30°C in SD without methionine and cysteine plus histidine and any other necessary supplements. Streak for single colonies onto SD medium supplemented with histidine and/or other necessary supplements

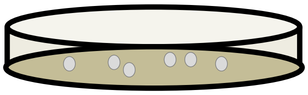

Days 12-13

Confirm genotype by PCR

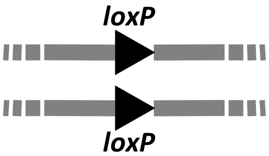

Days 13-14
