# Supplementary material for: New Clox Systems for Rapid and Efficient Gene Disruption in Candida albicans
Source: PLoS One. 2014 Jun 18;9(6):e100390. doi: 10.1371/journal.pone.0100390 (PMC4062495; doi:10.1371/journal.pone.0100390)
Supplement: Table S1 — Primers used in this study. (PDF) [file pone.0100390.s004.pdf]

Table S1. Primers used in this study.

Name	Use	Primer sequence (5' to 3')
Ade2∆-F2	*ade2* knockout cassette [1]	GCATATACAAGCACTACACATAATGGATAGCAAAACT GTTGGTATTTTAGGAGGTGGCCAATTAGGTCGTATG ATTGTTGAAGCAGCACAacggccagtgaattgtaata
Ade2∆-R2		CTATTTTTTCAATTTATCAGTTAAATAGTCTTCATATC CAATTTCTTCTAAAGTTTCTGCTTTACCTAATACTTCA GTTTCCATGTTCAAtcggaattaaccctcactaa
ADE2-ORF-F1	PCR diagnosis, wild type *ADE2* (outside ORF)	TCCACGACAAATAACGAACC
ADE2-ORF-R1		CTAAGAAGGGAAAAGCACC
ADE2-in-F1	PCR diagnosis, wild type *ADE2* (inside ORF)	TCCACGACAAATAACGAACC
ADE2-in-R1		ACCCATCTTTCGCTCTG
Ade2-NAT1-F	PCR diagnosis, *ade2-NAT1*	TCCACGACAAATAACGAACC
Ade2-NAT1- R		GTATATGTCTATGCCATGTCC
Ade2-cre-F1	PCR diagnosis, *ade2-cre*	TCCACGACAAATAACGAACC
Ade2-cre-R1		GATTCACATTGGTAGAACC
Ade2-ura-F2	PCR diagnosis, *ade2-ura3*	ATCCACGACAAATAACGAAC
Ade2-ura-R2		TCCCTGTTTTAATCCTTCAA
Ade2-his-F	PCR diagnosis, *ade2-his1*	ATCCACGACAAATAACGAAC
Ade2-his-R		CAATTTTCTTTCCAACAAGC
ACT1-F	PCR diagnosis, *ACT1* control	GTTGACCGAAGCTCCAATGAATCC
ACT1-R		CAGCAATACCTGGGAACATGG
GSH2∆-F1	*gsh2* knockout cassette [1]	ACAATCATCTAAGGAAAAAATGGGAAATAGCACTTTT GCTTACTGTACTAACCACATACATTCATTGCCAAGTC TTTGAACTACAAGAAAAcggccagtgaattgtaata
GSH2∆-R1		TATATGTTATCTACGCAGCCAAAACCGGCAGCAACT CCACCTTCATTGCTGTTACTGAATTTAGATCTCAACA ACCAACCAGCATTTTTGtcggaattaaccctcactaa
GSH2-ORF-F1	PCR diagnosis, wild type *GSH2* (outside ORF)	TGAATTGATTGTCTTGGTGA
GSH2-ORF-R1		AAACAGGAATCTCGTCTTCA
GSH2-in-F1	PCR diagnosis, wild type *GSH2* (intside ORF)	TGAATTGATTGTCTTGGTGA
GSH2-in-R1		TGACGCTATTCGTATCTGTG
Gsh2-ura-F1	PCR diagnosis, *gsh2-ura3*	TGAATTGATTGTCTTGGTGA
Gsh2-ura-R1		TCCCTGTTTTAATCCTTCAA
Clox-MET3p-F	*MET3p* Infusion cloning [2]	tagaagatctgctagCAATTGTCTATTCCAAGCCT
Clox-MET3p-R		aatgagtatacccggGGGGGAGGGTATTTACTTTTA
Clox-NAT1-F	*NAT1* Infusion cloning [2]	gatcaattcctgagcTCATCCCATTCATTCCATCA
Clox-NAT1-R		gactggtaagctagcGAAAGTATAGGAACTTCCATCAAGC
RPS1-NAT1-F	RPS1 cloning [3]	gcggcg<u>gagctc</u>TCGCGTAGATCCAACTCA
RPS1-NAT1-R		cgccgc<u>cccggg</u>TCTAGAACTAGTGGATCCCCCAGA

1. Upper case, homology to target locus; lower case, homology to *Clox* landing pad sequences.
2. Upper case, homology to target locus; lower case, homology for Infusion recombination.
3. Upper case, homology to *RPS1*; underline, restriction sites, lower case, additional non-homologous sequence.
